# A pitfall of thoracoscopic thymectomy: a case with intraoperative and postoperative complications

**DOI:** 10.1186/s40792-017-0374-3

**Published:** 2017-09-02

**Authors:** Motoki Yano, Hiroki Numanami, Masayuki Yamaji, Rumiko Taguchi, Chihiro Furuta, Masayuki Haniuda

**Affiliations:** Division of Chest Surgery, Department of Surgery, Aichi Medical University, 1-1 Yazakokarimata, Nagakute, 480-1195 Japan

**Keywords:** Thymectomy, Complication, Pericarditis, Bleeding

## Abstract

We have reported the usefulness of the subxiphoid approach in thymectomy. However, such a new operation method may have unknown complications that rarely occur. Surgeons cannot completely avoid intraoperative and postoperative complications. We report a case of intraoperative injury of the orifice of the left internal thoracic vein flowing to the left brachiocephalic vein and postoperative pericarditis following video-assisted thoracic surgery (VATS) thymectomy. The innominate vein has been considered to be the vessel that is most frequently injured especially at the orifice of the thymic veins. We also suggest that the orifice of the left internal thoracic vein is the second dangerous location that requires special care. In addition, postoperative pericarditis occurred in this patient. Pericardial drainage was necessary. No additional complications have been found in the 9 months since the operation. Though VATS thymectomy using the subxiphoid approach is a safe and less-invasive operation, intraoperative and postoperative complications were possible to be occurred.

## Background

We have reported the usefulness of the subxiphoid approach in thymectomy. However, we cannot completely avoid intraoperative and postoperative complications. We report a case of intraoperative injury of the orifice of the left internal thoracic vein flowing to the left brachiocephalic vein and postoperative pericarditis following VATS thymectomy.

## Case presentation

**Fig. 1:**
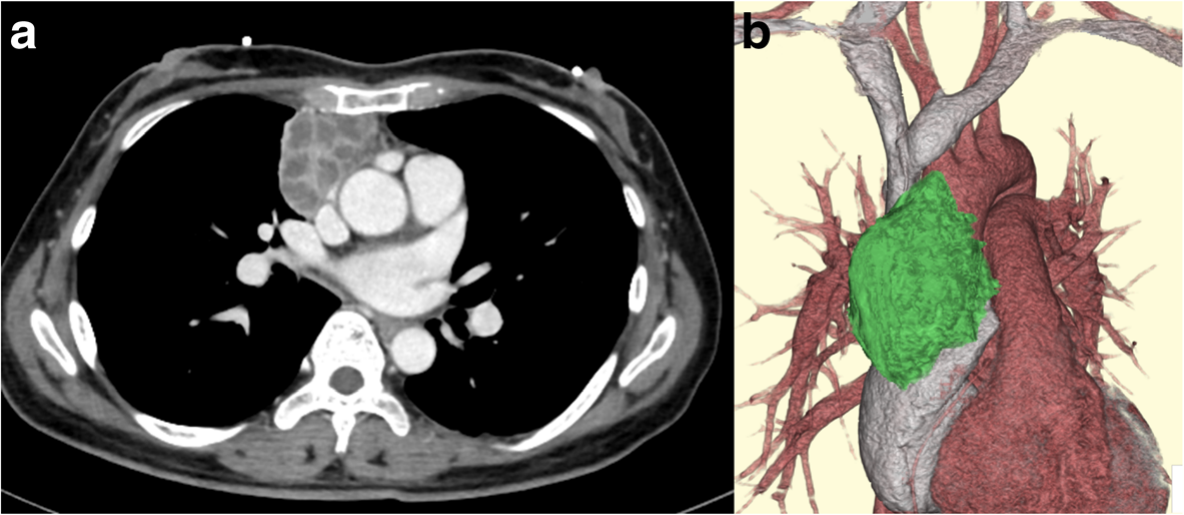
**a** An enhanced multiple cystic anterior mediastinal tumor. **b** A reconstructed three-dimensional image of the tumor and vessels

A 48-year-old woman visited our hospital with a chief complaint of chest pain. She had experienced similar symptoms 3 weeks previously. Chest computed tomography (CT) revealed an anterior mediastinal mass 7.0 cm in diameter enhanced with multiple cystic lesions (Fig. 1). Anti-acetyl-choline receptor autoantibodies were negative.

We selected a thoracoscopic operation. Thymectomy was initiated under general anesthesia with the patient in the lithotomy position using a single-lumen tracheal tube. Thoracoscopic thymectomy was performed as previously reported [1]. Dissection of the lateral edge of the thymus was performed using the LigaSure Maryland (Covidien, Mansfield, MA, USA) running along the phrenic nerve. The left side of the thymus was dissected after the right side. However, when the left upper edge of the thymus was dissected, bleeding suddenly occurred. Pressure was immediately applied to the bleeding point with an instrument. We confirmed that hemostasis had almost been obtained and thymectomy was continued. Following thymectomy, the bleeding point became obvious. We recognized a small tear in the orifice of the left internal thoracic vein flowing into the innominate vein. The amount of blood loss was only 10 g, but we were unable to suck the blood flowing into the bottom of the thoracic cavity completely. The total operation time was 165 min (Additional file 1).

**Fig. 2:**
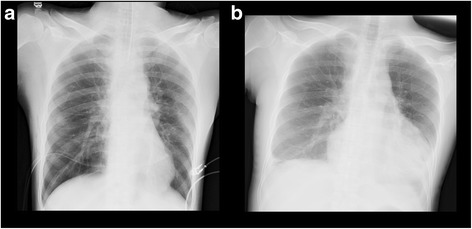
Postoperative chest radiography on the spine position: **a** immediately after the operation and **b** on the postoperative day 11. The cardiothoracic ratio was increased from 46.0 to 64.2%

The postoperative course was uneventful (Fig. 2a), and the chest drain was removed on the first postoperative day. The patient was discharged on the seventh postoperative day. However, this patient was readmitted on the 11th postoperative day because of palpitation and dyspnea. We diagnosed the patient with postoperative pericarditis (Fig. 2b), and 350 ml of diluted hemorrhagic effusion was drained. The patient was then discharged again. The anterior mediastinal tumor was diagnosed as a multilocular thymic cyst pathologically. Thereafter, the patient was diagnosed with Sjögren’s syndrome. No additional complications have been found in the 9 months since the operation.

## Discussion

**Fig. 3:**
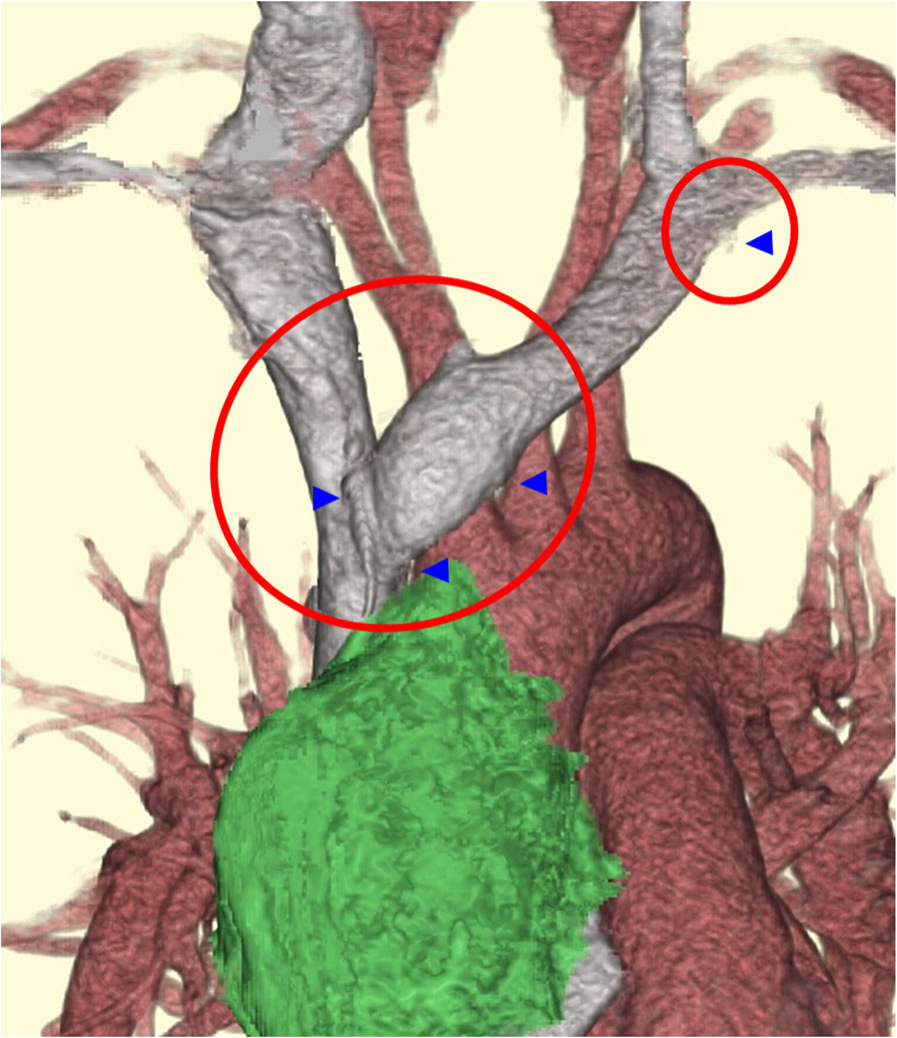
. The parts were easily injured during thoracoscopic thymectomy. Arrow heads indicate the orifices of the thymic veins and the internal thoracic vein

We recently established minimally invasive video-assisted thoracic surgery (VATS) thymectomy for anterior mediastinal tumors using the subxiphoid approach [1, 2]. However, such a new operation method may have unknown complications that rarely occur. VATS thymectomy has been regarded as a less-invasive method than thymectomy via sternotomy. A meta-analysis by Yang et al. indicated the benefits of VATS thymectomy in early stage thymomas [3]. Although a lower rate of intraoperative and postoperative complications has been reported in VATS thymectomy, the complications cannot be avoided entirely. Özkan and Toker reported a large series of VATS thymectomy, and they experienced catastrophic complications (1.5%) in 441 patients [4]. The innominate vein is the most frequent point of vessel injury. We usually pay particular attention when the dissection of the orifice of the thymic veins flows into the innominate vein (Fig. 3). We have newly found that the orifice of the left internal thoracic vein flows into the innominate vein, representing another point that can be easily torn during dissection.

We have not experienced intraoperative complications of vascular injury thus far. Özkan and Toker recorded at what number operation catastrophic complications occurred [4] and found points of occurrence ranging from the 26th to the 290th operation. We therefore endeavor to pay scrupulous attention during all operations, even if the operator is an expert, in order to avoid vessel injury during the operation.

We also experienced a rare complication of postoperative pericarditis. In a large series of VATS thymectomy, no cases of postoperative pericarditis were reported. Rowse et al. only reported two cases of postoperative pericarditis, and it was the second most frequent postoperative complication in his report [5]. Postoperative pericarditis might occur more frequently following sternotomy than VATS thymectomy [6]. The reason why postoperative pericarditis occurred in the present case was unclear. We compressed the bleeding point intraoperatively, and we might compress the underling heart. The cardiac compression might have an effect on the following pericarditis. We must ensure we take great care to avoid critical complications during and following surgery.

## Conclusions

The innominate vein is considered to be the vessel that is easily injured especially at the orifice of the thymic veins. Postoperative pericarditis is, also, considered to be postoperative complication.

## Additional file

Additional file 1: The right lobe and the lower edge of the left lobe of the thymus were already dissected. During dissection of the upper edge of the thymus, sudden bleeding occurred. Following the resection of the thymus and thymic tumor, the bleeding point was observed. We recognized a small tear in the orifice of the left internal thoracic vein flowing into the innominate vein.
